# HERC2/USP20 coordinates CHK1 activation by modulating CLASPIN stability

**DOI:** 10.1093/nar/gku978

**Published:** 2014-10-17

**Authors:** Min Zhu, Hongchang Zhao, Ji Liao, Xingzhi Xu

**Affiliations:** Beijing Key Laboratory of DNA Damage Response and College of Life Sciences, Capital Normal University, Beijing 100048, China

## Abstract

CLASPIN is an essential mediator in the DNA replication checkpoint, responsible for ATR (ataxia telangiectasia and Rad3-related protein)-dependent activation of CHK1 (checkpoint kinase 1). Here we found a dynamic signaling pathway that regulates CLASPIN turn over. Under unperturbed conditions, the E3 ubiquitin ligase HERC2 regulates the stability of the deubiquitinating enzyme USP20 by promoting ubiquitination-mediated proteasomal degradation. Under replication stress, ATR-mediated phosphorylation of USP20 results in the disassociation of HERC2 from USP20. USP20 in turn deubiquitinates K48-linked-polyubiquitinated CLASPIN, stabilizing CLASPIN and ultimately promoting CHK1 phosphorylation and CHK1-directed checkpoint activation. Inhibition of USP20 expression promotes chromosome instability and xenograft tumor growth. Taken together, our findings demonstrated a novel function of HERC2/USP20 in coordinating CHK1 activation by modulating CLASPIN stability, which ultimately promotes genome stability and suppresses tumor growth.

## INTRODUCTION

Our genome is extremely vulnerable in S-phase when the genetic material is being duplicated and in mitotic phase when the pairs of sister chromatids are being equally separated into two daughter cells (1,2). During S-phase, the progression of replication forks is often impeded by various forms of exogenous and endogenous DNA damage (3). When the replication fork progression is halted, the intra-S-phase checkpoint is activated, promoting structural stability of stalled forks and preventing the replisome components from dissociation (4,5). This ensures the rapid resumption of replication following DNA repair. The ATR (ataxia telangiectasia and Rad3-related protein)-CHK1 (checkpoint kinase 1) pathway plays key roles in activating the intra-S-phase checkpoint and in stabilizing the stalled replication forks (5–8).

The ATR-CHK1 pathway responds principally to single-strand DNA (ssDNA). ssDNA is coated by replication protein A and sensed by the complex of ATR and ATRIP (ATR-interacting protein) (9–11). The ATR/ATRIP complex, in coordination with RAD17 and the 9–1–1 (RAD9-HUS1-RAD1) complex, phosphorylates CHK1 on serines 317 and 345 and activates it on chromatin in a CLASPIN-dependent manner. Fully activated CHK1 is then released from chromatin and phosphorylates downstream effectors (3,5,11–12).CLASPIN is a critical mediator in the DNA replication checkpoint, responsible for ATR-dependent activation of CHK1 (12–14). Its expression is high in S and G2 phases, and declines sharply upon entry into mitosis and throughout G1 (15). In the G1 phase, CLASPIN is degraded by the APC^Cdh1^-mediated K48-linked polyubiquitination, whereas the ubiquitin-specific processing protease USP28-mediated deubiquitination prevents its degradation (16). At the onset of mitosis CLASPIN is degraded by SCF^bTrCP^-mediated ubiquitination, whereas USP7-mediated deubiquitination prevents its degradation (17). It has been demonstrated that the breast cancer suppressor, BRCA1, forms a complex with CLASPIN regulating CHK1 activation during replication (18,19). In addition to its association with BRCA1 and CHK1, CLASPIN also binds specifically to branched DNA structures and may associate with S-phase chromatin following formation of the pre-replication complex. This suggests that CLASPIN may play a role in monitoring the integrity of DNA replication forks. A recent report demonstrates that BRCA1-mediated K6-linked polyubiquitination of CLASPIN is required for efficient chromatin loading, but the corresponding deubiquitinases (DUBs) is not identified yet (19). Furthermore, how CLASPIN stability during S-phase is maintained is not yet elucidated either.HERC2, a large HECT domain-containing E3 ubiquitin ligase, is essential for DNA damage repair pathways, including homologous recombination repair of DNA double-strand breaks (DSBs) in particular. It interacts with another E3 ubiquitin ligase RNF8, coordinating the ubiquitin-dependent assembly of DNA repair factors on the damaged sites (20,21). In addition, HERC2 is a component of the DNA replication fork complex. It interacts with CLASPIN in the presence of BRCA1, regulating DNA origin firing and replication fork progression (22).

The DUB USP20 mediates removal of both K48- and K63-linked polyubiquitin chains. It has been shown to regulate G-protein coupled receptor signaling by deubiquitination of beta-2 adrenergic receptor (23,24). USP20 also deubiquitinates hypoxia-inducible factor-1 alpha (HIF-1α), promoting HIF-1α stability and consequently the expression of its target genes (25,26).

In this study, we have uncovered that HERC2/USP20 controls CLASPIN stability, modulating CHK1 activation in response to replication stress.

## MATERIALS AND METHODS

### Reagents, antibodies, expression constructs and cell lines

Hydroxyurea (HU, a final concentration of 2 mM was used throughout this study), cycloheximide (CHX, a final concentration of 50 μg/ml was used) and the ATR inhibitor NU6027 (a final concentration of 10 μM was used), were purchased from Sigma. BrdU (a final concentration of 20 μM was used) was from BD Biosciences.

Rabbit polyclonal antibodies used for immunoblotting and/or immunoprecipitaion including anti-MYC (A190–205A), anti-HA (A190–208A), anti-USP20 (A301–189A), anti-CLASPIN (A300–267A), anti-HERC2 (A301–905A), anti-ATR (A300–138A) were from the Bethyl Laboratories; Chk1 antibody (sc-8408) was from the Santa Cruz Biotechnology. Rabbit monoclonal antibody anti-GST (A00865) was from the GenScript. Mouse monoclonal antibody anti-FLAG M2 (F1804) was from Sigma. Phospho-Chk1 (Ser345) (Rabbit mAb #2348) and Phospho-(Ser/Thr) ATM/ATR substrate antibody (#2851) were from Cell Signaling. Anti-BrdU fluorescein isothiocyanate (FITC) (347583) used for immunofluorescence was from BD Biosciences.

The detailed information of all the expression constructs is available upon request.

All cell lines were cultured in high-glucose Dulbecco's modified Eagle's medium supplemented with 10% fetal bovine serum at 37°C.

### siRNA transfections and RNA interference

The siRNA oligonucleotide duplexes against USP20 (si1-USP20 sequence: CCATAGGAGAGGTGACCAA; si2-USP20 sequence: GGACAATGATGCTCACCTA), HERC2 (si1-HERC2 sequence: GCGGAAGCCTCATTAGAAA; si2-HERC2 sequence: GAGCTGATTTCTTGAGTAA) and the non-target siRNA control (siCTR) were purchased from Guangzhou RiboBio. Cells were transfected with siRNA oligonucleotide complexes at a final concentration of 20 nM using RNAimax (Invitrogen) according to the manufacturer's instructions. The shCTR and sh-USP20 (target sequence: ACACCTTCATCAAGTTGAA) lentiviral particles were purchased from Shanghai GeneChem.

### Immunoprecipitation and immunoblotting

Immunoprecipitation and/or immunoblotting were performed essentially as described before (27).

### BrdU incorporation assay

Mock- or USP20-depleted A549 cells were irradiated with ultraviolet (UV) (20 J/m^2^) and pulse-labeled 40 min later with BrdU at a final concentration of 20 μM for 20 min. Cells were then washed with phosphate buffered saline (PBS) twice and fixed with 75% ethanol (vol/vol) at room temperature for 30 min. After washing with PBS, the fixed cells were incubated with 2M HCl at room temperature for 30 min, neutralized with 0.1 M Na2B4O7 for 10 min. Cells on cover-slips were blocked with 2% bovine serum albumin in PBS with 0.1% Tween 20 (PBST) for 30 min and then incubated with FITC-conjugated anti-BrdU antibody (1:50) at room temperature for 60 min. Cells were stained with 4′,6-diamidino-2-phenylindole (DAPI) for 2 min after extensive washing with PBST. The cover-slips were mounted onto glass slides with anti-fade solution and visualized using a fluorescence microscope.

### Deubiquitination assays

For *in vivo* deubiquitination assays for CLASPIN, 293T cells were co-transfected with MYC-USP20 or MYC-USP20(C154S), FLAG-CLASPIN and HA-Ub (K48 only, K63 only or K6 only), cells were collected 48 h after transfection, washed in PBS once, cell extracts were subjected to immunoprecipitation with as immunoprecipitation procedure and immunoblotting with antibodies as indicated.

For *in vitro* deubiquitination assays for CLASPIN, 293T cells were co-transfected with FLAG-CLASPIN and HA-UB. Total cell lysates were harvested 2 days later in NETN buffer (20 mM Tris-HCL pH8.0, 100 mM NaCl, 1 mM ethylenediaminetetraacetic acid, 0.5% NP-40, and protease inhibitor cocktail) for immunoprecipitation with anti-FLAG agarose beads (Sigma). The FLAG-CLASPIN immunocomplex was incubated with bacterially produced GST, GST-USP20 or GST-USP20(C154S) in the deubiquitination buffer (50 mM Tris-HCl pH 8.0, 50 mM NaCl, 1 mM ethylenediaminetetraacetic acid, 10 mM DTT, 5% glycerol) (28) at 37°C for 2 h and then subjected for immunoblotting.

### Mitotic chromatin spreads

The mitotic chromatin spreads for mock- or USP20-depleted A549 cells were prepared essentially as described before (29). More than 80 spreads were randomly selected for analysis.

### HCT116 cell tumor xenografts

Eighteen female BALB/c nude mice (6–8 weeks old with a body weight of 18–22 g) were randomly assigned to two groups, 9 mice per group. Note that 5 × 10^6^ mock-depleted (shCTR) or USP20-depleted (sh-USP20) HCT116 cells were injected subcutaneously into the right lower flanks of mice. The tumor volume was measured in two dimensions using a caliper (recorded up to one decimal point) starting from day 9 after injection and ending at day 51 at a frequency of twice a week. Tumor volume, expressed in mm^3^, was calculated using the following formula, in which ‘*a*’ and ‘*b*’ are the long and the short diameters of a tumor, respectively. *V* (mm^3^) = (*a* × *b*^2^)/2. Experiments were approved by the Institutional Animal Care and Use Committee (CLS20130113).

## RESULTS

### USP20 promotes CHK1 activation in response to replication stress or UV radiation

**Figure 1.**
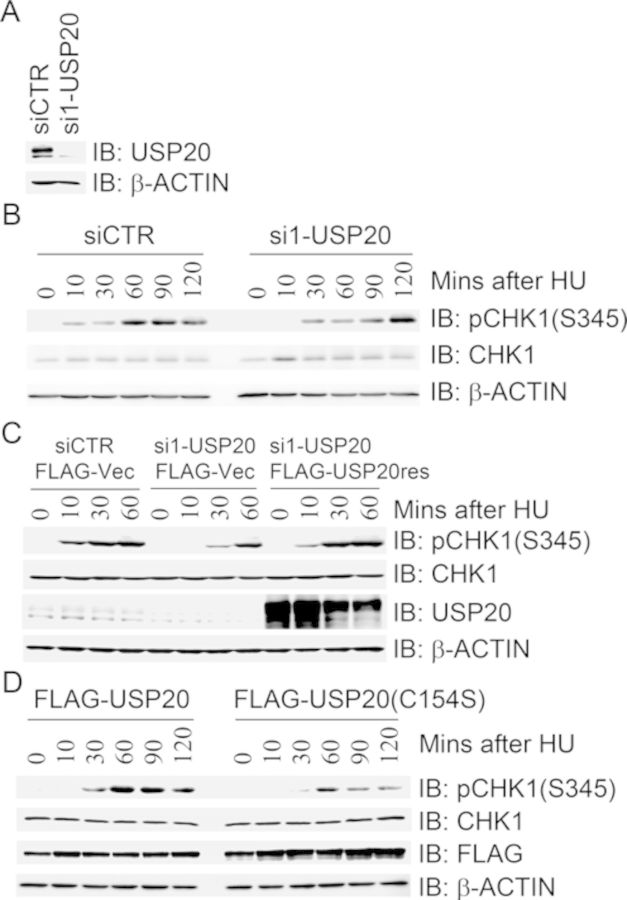
USP20 promotes CHK1 activation in response to replication stress. (A and B) Depletion of USP20 delayed HU-induced CHK1 activation. 293T cells were transfected with a control siRNA (siCTR) or a USP20-specific siRNA (si1-USP20), cells were treated with HU 2 days after transfection. Total cell lysates were harvested at different time points after HU treatment and used for immunoblotting with antibodies as indicated. The efficiency of USP20 knockdown was determined by immunoblotting with antibodies against USP20 and β-actin (A). (C) Expression of siRNA-resistant form of USP20 rescued USP20-depletion-induced delay of CHK1 activation in response to HU treatment. 293T cells were transfected with siCTR or si1-USP20. USP20-depleted cells were introduced 48 h after the first transfection with FLAG vector or FLAG-USP20res, the transfectants were treated 2 days later with HU at different time points. Total cell lysates were harvested and used for immunoblotting with antibodies as indicated. (D) Overexpression of the catalytically inactive mutant USP20(C154S) attenuated HU-induced CHK1 activation. 293T cells were transfected with FLAG-USP20 or FLAG-USP20(C154S). Cells were treated 2 days later with HU at different time points. Total cell lysates were harvested and used for immunoblotting with antibodies as indicated.

In a candidate screen of ubiquitin-specific processing proteases (USPs) for modulating CHK1 activation, we revealed that inhibition of USP20 expression in 293T cells delayed HU-induced CHK1 activation (Figure 1A and B), whereas expression of siRNA-resistant form of FLAG-USP20res in the endogenous USP20-depleted cells restored HU-induced CHK1 activation kinetics (Figure 1C). The faster migration form of USP20 is likely a non-specific signal because it was not greatly reduced in comparison with the slower migration form upon USP20 depletion with three independent siRNA oligonucleotide duplexes (Figure 1A and data not shown). Concomitantly, expression of the catalytically inactive mutant FLAG-USP20(C154S) attenuated HU-induced CHK1 activation (Figure 1D). We also found that either inhibition of USP20 expression or expression of the catalytically inactive mutant FLAG-USP20(C154S) led to a decrease of pCHK1(S345) levels 30 min after UV treatment unlike the corresponding controls (Supplementary Figure S1). Taken together, these results indicate that USP20 promotes CHK1 activation in response to HU-induced replication stress or UV irradiation.

### USP20 promotes CLASPIN stability through deubiquitination

**Figure 2.**
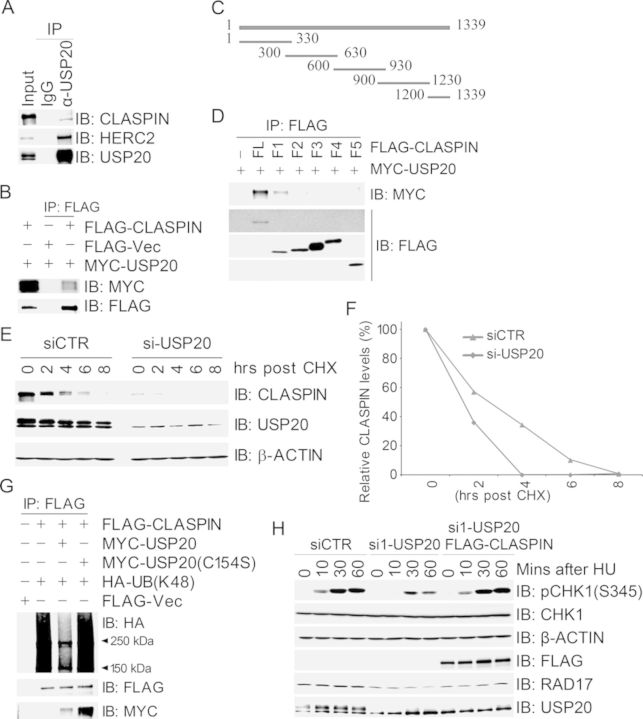
USP20 promotes CLASPIN stability through deubiquitination. (A and B) USP20 interacted with CLASPIN. Total cell lysates were extracted from 293T cells (A) or 293T cells co-transfected with MYC-USP20 and FLAG-CLASPIN (B), and subjected to immunoprecipitation and immunoblotting with antibodies as indicated. (C) Schematic structure of CLASPIN fragments. (D) The N terminus of CLASPIN (1–330 AAs) mediated the interaction with USP20. Total cell lysates extracted from 293T cells co-transfected with MYC-USP20 and FLAG-CLASPIN or its fragments were subjected to immunoprecipitation with an anti-FLAG antibody followed by immunoblotting with an anti-MYC antibody. (E and F) Inhibition of USP20 expression decreased CLASPIN protein levels and shortened CLASPIN half-life. The siCTR- or si-USP20-transfected 293T cells were treated with CHX at different time points. Total cell lysates were harvested and used for immunoblotting with antibodies as indicated in (E), and quantification of the CLAPSIN/β-ACTIN ratio was plotted in (F). (G) USP20 deubiquitinated K48-linked polyubiquitination of CLASPIN *in vivo*. 293T cells were co-transfected with the expression constructs as indicated, total cell lysates were harvested 2 days later and subjected to immunoprecipitation followed by immunoblotting with antibodies as indicated. (H) USP20 depletion-induced delay of CHK1 activation in response to HU treatment was reversed by overexpression of CLASPIN. USP20-depleted 293T cells were transfected with FLAG-VEC or wild-type FLAG-CLASPIN and treated 2 days later with HU at different time points. Total cell lysates were extracted and used for immunoblotting with antibodies as indicated.

CLASPIN is an adapter protein which binds to CHK1 and facilitates the ATR-dependent phosphorylation and activation of CHK1 (14,30). We thus reasoned that USP20 may have a functional link with CLASPIN and/or CHK1. Co-immunoprecipitation assays revealed that endogenous CLASPIN in 293T cells was present in the endogenous USP20 immunocomplex (Figure 2A), and MYC-USP20 was present in the FLAG-CLASPIN immunocomplex when both were expressed in 293T cells (Figure 2B). We failed to detect USP20 in the anti-CHK1 immunocomplex or CHK1 in the anti-USP20 immunocomplex (data not shown). We generated six truncated fragments spanning the full-length CLASPIN polypeptide with limited overlap (Figure 2C), and domain mapping experiments uncovered that the N-terminus of CLASPIN (1–330 AAs) mediated the interaction with USP20 (Figure 2D).

We then wanted to determine the functional significance of the interaction between USP20 and CLAPSIN. Inhibition of USP20 expression by siRNA resulted in a dramatic decrease of CLASPIN protein levels (Figure 2E), and, when cells were treated with CHX, an inhibitor of protein biosynthesis, CLASPIN protein levels in USP20-depleted cells decreased faster than those in mocked-depleted cells (Figure 2E and F). Concomitantly, expression of the catalytically inactive mutant FLAG-USP20(C154S) reduced the half-life of CLASPIN (Supplementary Figure S2). Furthermore, expression of wild-type MYC-USP20, but not MYC-USP20(C154S), reduced K48-linked, but not K6- or K63-linked polyubiquitination of CLASPIN in 293T cells (Figure 2G and Supplementary Figure S3). CLASPIN was also deubiquitinated by bacterially produced GST-USP20, but not by the catalytically inactive mutant GST-USP20(C154S), in the *in vitro* deubiquitination assays (Supplementary Figure S4). Taken together, these data suggest that USP20 mediates deubiquitination of K48-linked polyubiquitination of CLASPIN, promoting its stability.

To test if USP20-mediated stability of CLASPIN promotes CHK1 activation upon replication stress, we expressed wild-type FLAG-CLASPIN in USP20-depleted cells. We found that overexpression of FLAG-CLASPIN corrected the delay of USP20 depletion-induced CHK1 activation in response to HU treatment (Figure 2H). It was also noted that inhibition of USP20 expression decreased RAD17 protein levels (Figure 2H). This is consistent with the observation by Shaheen *et al.* (31). Given that USP20 depletion-induced promotion of CHK1 activation is rescued by overexpression of CLASPIN (Figure 2H), CLASPIN is the major substrate for USP20 in CHK1 activation.

### HERC2 promotes USP20 degradation

**Figure 3.**
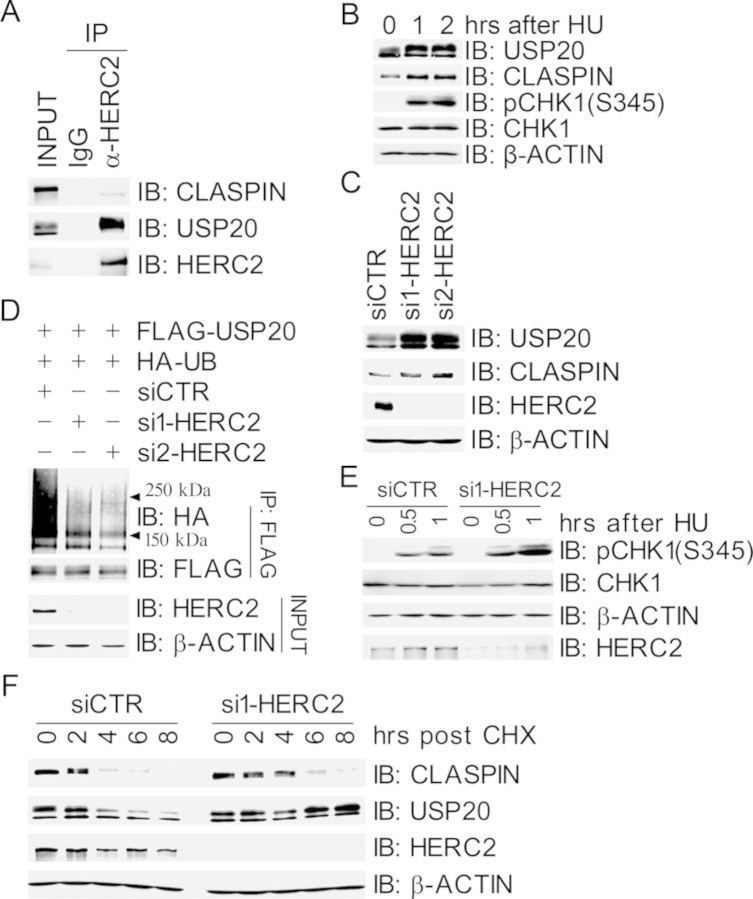
HERC2 promotes USP20 ubiquitination and subsequent degradation. (A) USP20 interacted with HERC2. Total cell lysates were extracted from 293T cells and subjected to immunoprecipitation and immunoblotting with antibodies as indicated. (B) Both USP20 and CLASPIN protein levels increased in response to HU treatment. 293T cells were treated with HU at different time points as indicated, total cell lysates were extracted and subjected to immunoblotting with antibodies as indicated. (C) Inhibition of HERC2 expression increased protein levels of USP20 and CLASPIN. 293T cells were transfected with siCTR or HERC2-specific siRNA (si1-HERC2 and si2-HERC2). Total cell lysates were harvested 2 days later and subjected to immunoblotting with antibodies as indicated. (D) Inhibition of HERC2 expression decreased the ubiquitination levels of FLAG-USP20. HERC2-depleted 293T cells were co-transfected with expression constructs of FLAG-USP20 and HA-UB. Total cell lysates were harvested 2 days later and subjected to immunoprecipitation and immunoblotting with antibodies as indicated. (E) Depletion of HERC2 promotes HU-induced CHK1 activation. HERC2-depleted 293T cells were treated with HU at different time points as indicated. Total cell lysates were harvested and subjected to immunoblotting with antibodies as indicated. (F) Inhibition of HERC2 expression prolonged the half-life of USP20 and CLASPIN. Mocked- or HERC2-depleted 293T cells were treated with CHX at different time points as indicated, total cell lysates were harvested and subjected to immunoblotting with antibodies as indicated.

We next wanted to investigate how USP20 is regulated in response to replication stress. It has been reported that the E3 ligase HERC2 is a component of the replication fork complex that interacts with CLASPIN (22), however, the functional relationship between HERC2 and CLASPIN remains unclear. Co-immunoprecipitation assays revealed that both HERC2 and CLASPIN were present in the USP20 immunocomplex (Figure 2A) and both USP20 and CLASPIN were in the HERC2 immunocomplex (Figure 3A), suggesting that HERC2, USP20 and CLASPIN form a complex. Domain mapping revealed that the F4 fragment of HERC2 (2600–3600 AAs) mediated the interaction with USP20 (Supplementary Figure S5). This fragment contains the ZZ domain with a SUMO-binding motif, mutation of which severely compromises RNF8 binding (21). We found that both USP20 and CLASPIN protein levels in 293T cells increased after HU treatment (Figure 3B). Furthermore, the residual levels of CLASPIN after HU treatment were not increased in USP20-depleted cells (Supplementary Figure S6), indicating that the increase of CLASPIN after HU treatment may be controlled by USP20.

Given that HERC2 is an E3 ligase involved in the DNA damage response, we speculated if USP20 is a substrate of HERC2. Indeed, inhibition of HERC2 expression using two independent siRNA oligos in 293T cells resulted in an increase of protein levels of both USP20 and CLASPIN (Figure 3C) and a decrease of ubiquitination levels of FLAG-USP20 (Figure 3D). Depletion of HERC2 in 293T cells also enhanced CHK1 activation upon HU treatment (Figure 3E). Furthermore, depletion of HERC2 stabilized USP20, thus prolonging the half-life of CLASPIN (Figure 3F). We next attempted to test if expression of siRNA-resistant form of HERC2 would rescue HERC2 depletion-induced phenotypes. We repeatedly failed to express an epitope-tagged version of full-length HERC2, this could be due to the fact that human HERC2 encodes a huge polypeptide of 4834 amino acids. Nevertheless, we engineered a fusion of the F4 fragment, which mediates the interaction with USP20, and the F6 fragment, which is sufficient for its E3 ligase activity (32). This fusion and its catalytically inactive mutant physically interacted with USP20 (Supplementary Figure S7A). However, expression of this fusion did not have an impact on USP20 stability (Supplementary Figure S7B) and HU-induced CHK1 activation (Supplementary Figure S7C) in HERC2-depleted cells. This indicates that additional elements within HERC2 are required for maintenance of USP20 stability by HERC2. Taken together, these results demonstrated that HERC2 destabilized USP20 through ubiquitination and subsequent degradation.

### ATR-mediated phosphorylation of USP20 promotes its dissociation from HERC2

**Figure 4.**
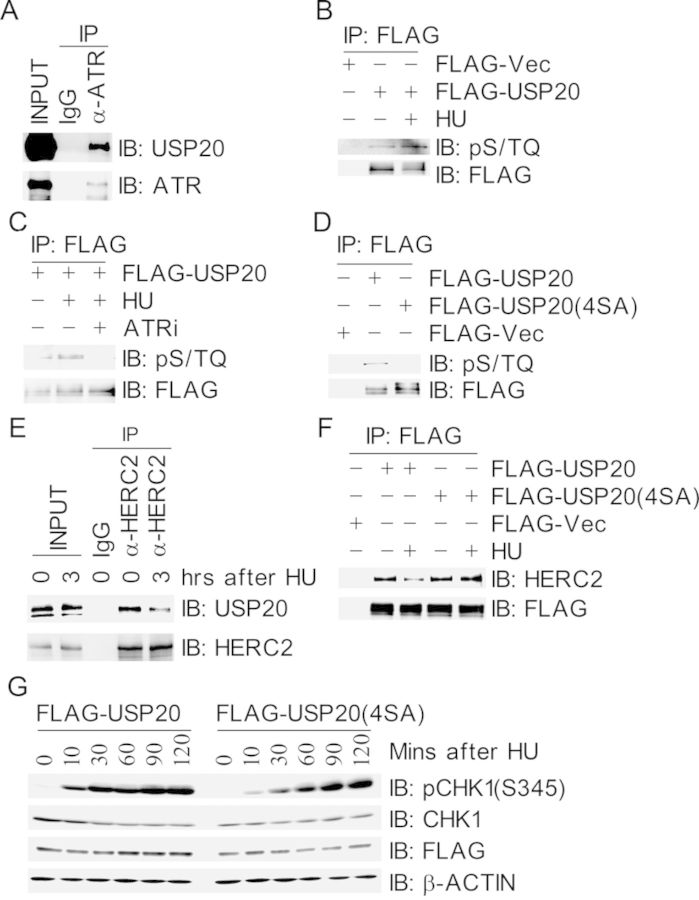
ATR-mediated phosphorylation of USP20 promotes its dissociation from HERC2. (A) USP20 interacted with ATR. Total cell lysates were extracted from 293T cells and subjected to immunoprecipitation followed by immunoblotting with antibodies as indicated. (B and C) USP20 was phosphorylated by ATR in response to HU treatment. 293T cells expressing FLAG-USP20 were mocked-treated or treated with HU for 3 h (B) or pre-treated with the ATR inhibitor NU6027 (ATRi) for 1 h before HU treatment (C), total cells lysates were harvested and subjected to immunoprecipitation followed by immunoblotting with antibodies as indicated. (D) USP20 was phosphorylated on its S/TQ motifs. 293T cells were transfected with FLAG-VEC, FLAG-USP20 or FLAG-USP20(4SA), in which all the four S/TQ sites within the USP20 polypeptide were mutated to AQ. Total cells lysates were harvested 2 days later and subjected to immunoprecipitation followed by immunoblotting with antibodies as indicated. (E) The interaction between HERC2 and USP20 decreased under replication stress. Total cell lysates were extracted from 293T cells with mock treatment or HU treatment for 3 h and immunoprecipitated with an anti-HERC2 antibody. Immunoprecipitates were subjected to immunoblotting with antibodies as indicated. (F) Phosphorylation-deficient USP20(4SA) stably associated with HERC2 after HU treatment. 293T cells expressing FLAG-USP20 or FLAG-USP20(4SA) were treated with HU for 3 h, total cells lysates were harvested and subjected to immunoprecipitation followed by immunoblotting with antibodies as indicated. (G) Phosphorylation-deficient mutant USP20(4SA) delayed HU-induced CHK1 activation. 293T cells expressing FLAG-USP20 or FLAG-USP20(4SA) were treated with HU at different time points as indicated, and total cell lysates were extracted and used for immunoblotting with antibodies as indicated.

Since USP20 is upregulated after replication stress, we sought to determine whether USP20 is modulated by ATR in response to replication stress. Co-immunoprecipitation assays revealed that USP20 was present in the ATR immunocomplex (Figure 4A). The ATM/ATR substrate pS/TQ antibody was reactive with the immunoprecipitated FLAG-USP20 under unperturbed conditions, and this reactivity increased upon HU treatment (Figure 4B), whereas this reactivity was diminished when cells were pretreated with the ATR-specific inhibitor NU6027 (Figure 4C). Furthermore, the pS/TQ antibody was not reactive with the immunoprecipitated FLAG-USP20 (4SA), in which all the four SQ sites were mutated into alanine (Figure 4D). Taken together, these data demonstrated that USP20 is phosphorylated by ATR in response to HU-induced replication stress.

We then wanted to determine the biological function of ATR-mediated phosphorylation of USP20. We found that the presence of USP20 in the HERC2 immunocomplex decreased upon HU treatment (Figure 4E), so did the presence of HERC2 in the FALG-USP20 immunocomplex, whereas the interaction between USP20(4SA) and HERC2 did not decrease in response to HU treatment (Figure 4F). These suggest that ATR-mediated phosphorylation of USP20 promotes dissociation of USP20 from HERC2, and this dissociation would stabilize CLASPIN and promote CHK1 activation. Indeed, when cells were treated with CHX, the turnover of CLASPIN became slower in the FLAG-USP20(4SA) expressing cells than that in the FLAG-USP20-expressing cells (Supplementary Figure S8). Concomitantly, both HU-induced (Figure 4G) and UV-induced (Supplementary Figure S9) CHK1 activation slowed down in FLAG-USP20(4SA)-expressing cells when compared with that in FLAG-USP20-expressing cells (Figure 4G).

### USP20 promotes genome stability and suppresses xenograft tumor growth

**Figure 5.**
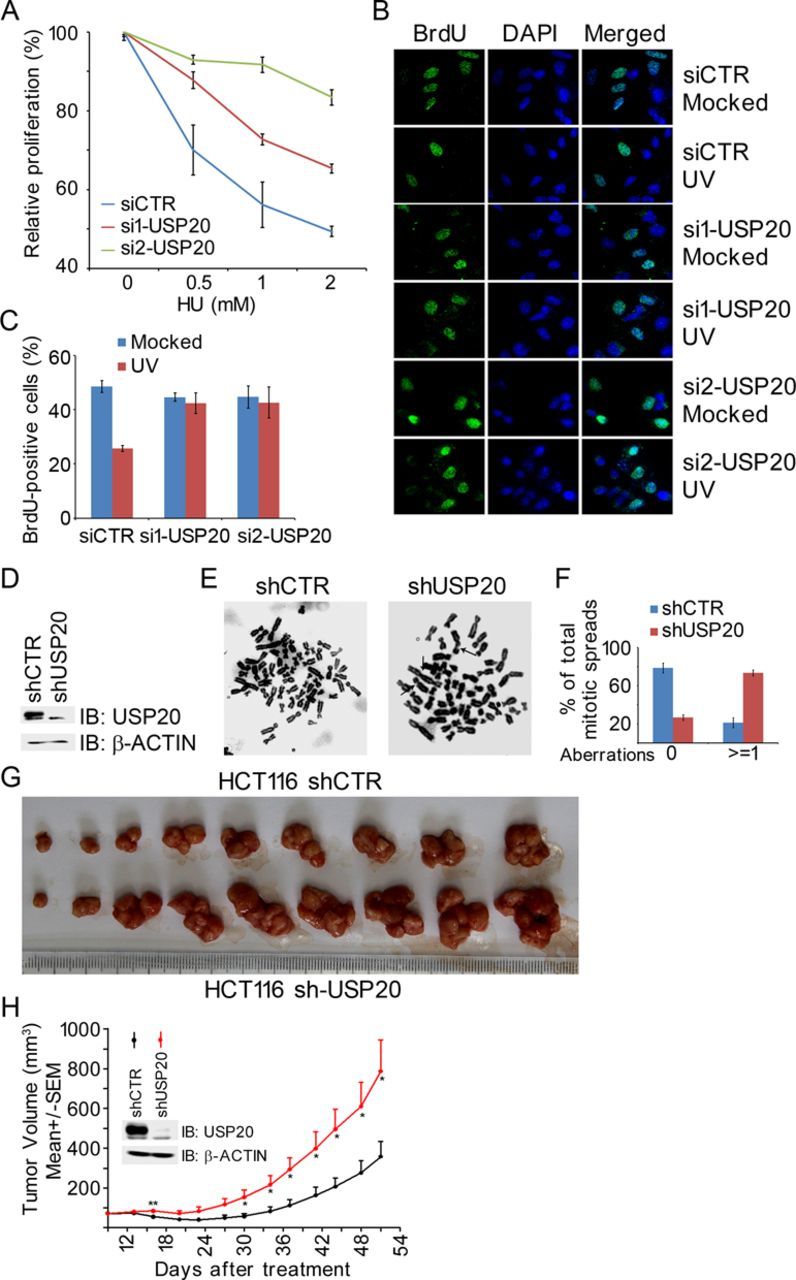
USP20 promotes genome stability and suppresses xenograft tumor growth. (A) Depletion of USP20 promotes cell proliferation. A549 cells were transfected with siCTR, si1-USP20 or si2-USP20, transfectants were split 2 days later into a 96-well plate (5000 cells per well) and treated 16 h later with different doses of HU for 72 h. Cell proliferation was determined by the MTT assay. The USP20 knockdown efficiency is shown in Supplementary Figure S10. (B and C) Depletion of USP20 compromised UV-induced intra-S-phase checkpoint. Mock- or USP20-depleted A549 cells described in panel (A) were UV irradiated with a dose of 20 J/m^2^, pulse-labeled 40 min later with BrdU for 20 min, fixed with 75% ethanol and stained with an anti-BrdU antibody and DAPI (B). BrdU-positive cells in randomly selected fields (more than 100 cells per treatment) were quantified in (C). Three independent experiments were performed. (D–F) USP20 depletion resulted in chromosome aberrations. A549 cells with mocked- or shRNA-mediated USP20 depletion (D) were subjected to a mitotic spread assay. Representative metaphase spreads were shown in (E) and the arrows pointed to the chromosome aberrations. A histogram summarizing percentages of spreads containing aberrant chromosomal structures was shown in (D). More than 80 spreads were randomly selected for analysis. Three independent experiments were performed. (G and H) USP20 suppressed xenograft tumor growth. Note that 5 × 10^6^ mock-depleted (shCTR) or USP20-depleted (sh-USP20) HCT116 cells were injected subcutaneously into the right lower flanks of BALB/c nude mice. The tumor volume was measured starting from day 9 after injection and ending at day 51 at a frequency of twice a week. Tumor volumes were shown as mean ± SEM, and the Student's *t*-test was performed. Tumor images were shown in (G), while quantification results were shown in (H). *P < 0.05.

Our results demonstrated that USP20 positively regulates the ATR-CLASPIN-CHK1 signaling during replication stress. We thus attempted to determine the biological significance of USP20. It was found that inhibition of USP20 expression by two independent siRNA oligos in A549 cells became less sensitive to HU treatment in the cell proliferation assays (Figure 5A). When A549 cells were UV-irradiated and pulse-labeled 40 min later with BrdU for 20 min, about 50% of mock-treated cells were BrdU positive, UV treatment reduced the percentage of BrdU-positive cells to about 30%, whereas this reduction disappeared in USP20-depleted cells in response to UV treatment (Figure 5B and C), indicating that USP20 is required for UV-induced activation of the intra-S-phase checkpoint.

With a defective intra-S-phase checkpoint in the USP20-depleted cells, we would expect that these cells would exhibit genome instability leading to enhanced tumor growth. Indeed, mitotic spread experiments revealed that inhibition of USP20 expression in A549 cells induced significantly more chromosomal aberrations, including fragmented chromosomes, detached/multiple centromeres and gaps (Figure 5D and E). Furthermore, in a mouse xenograft tumor model, colon cancer HCT116 cells stably depleted of USP20 expression by lentiviral shRNA, when transplanted onto nude mice, developed significantly larger tumor volumes than mock-depleted HCT116 cells (Figure 5G and H). These results suggest that USP20 is a possible tumor suppressor.

## DISCUSSION

In addition to BRCA1-mediated K6-linked polyubiquitination of CLASPIN and an unknown DUB-mediated removal of K6-linked polyubiquitin chains of CLASPIN, both of which coordinate chromatin loading of CLASPIN and subsequent CHK1 activation (19), the stability of CLASPIN is important for CHK1 activation as well. CLASPIN protein levels fluctuate throughout the cell cycle, as the opposing actions of APC^Cdh1^/USP28 and SCF^bTrCP^/USP7 ensures low CLASPIN protein levels during the G1 phase and the mitotic phase, respectively (15–17). Given that HERC2 is a component of the replication complex, our data shown in this report suggest that HERC2/USP20 coordinately modulate CLASPIN stability during S-phase and in response to replication stress. Under unperturbed condition, HERC2 ubiquitinates USP20 and promotes ubiquitination-mediated proteasomal degradation of USP20, regulating the status of K48-linked polyubiquitination of CLASPIN and ensuring appropriate protein levels of CLASPIN during the S-phase. It warrants further investigation to determine if HERC2 directly ubiquitinates CLASPIN and its subsequent degradation. Upon replication stress, ATR-mediated phosphorylation of USP20 promotes dissociation of HERC2 from USP20, stabilizes USP20 and its association with CLASPIN, thus increasing CALSPIN stability and ensuring CHK1 activation.

In addition to USP20, a few other USPs modulate CLASPIN stability and subsequent CHK1 activation in response to UV and/or HU treatment. Inhibition of USP7 expression destabilizes both CLASPIN and CHK1, compromising CHK1 activation in response to UV irradiation or HU treatment (17). It has been reported that USP28 stabilizes CALSPIN; however, the significance of this stabilization in DNA Damage Response (DDR) is not clear yet (16). A recent report indicates that USP29 promotes CLASPIN (not CHK1) stability, ensuring CHK1 activation in response to UV irradiation (33). It would be extremely intriguing and challenging to tease out how these USPs coordinate to control CLASPIN stability throughout the cell cycle under unperturbed condition and ensure appropriate checkpoint activation in response to replication stress.K48- and K63-linked polyubiquitin chains have been detected seconds after DNA damage. The first E3 ligase so far recruited to the DSB site is CHFR (checkpoint with forkhead and RING finger domains protein) and this recruitment is poly (ADP-ribose)-dependent, leading to the first wave of ubiquitination events involved in the initial stage of the DDR (34). To ensure the appropriate ubiquitination status (as well as other post-translational modification status) of DDR proteins on and/or being recruited to the damaged sites, it is logical to assume that deubiquitination events occur at the early stage of DDR. Alternatively, reversible ubiquitination events may occur actively at the very early stage of DDR and fine-tuning of ubiquitination status of key DDR factors may ensure appropriate checkpoint activation and ultimately promote genome stability. Regulation of CLASPIN stability by several E3 ligase/DUB pairs is an example of such mechanisms (15,19).

Several cancer genomics studies have identified USP20 mutation in a variety of solid tumors, including colorectal adenocarcinoma with a mutation frequency of 5.6% (4 out of 72 cases) (35), uterine corpus endometrioid carcinoma with 3.8% of cases mutated (9 out of 240 cases) (36) and bladder urothelial carcinoma with a mutation frequency of 3.1% (4 out of 127 cases) (37). Our study as well as those of others’ has demonstrated that USP20 may ensure genome stability and suppress xenograft tumor growth. Taken together, these findings suggest that USP20 may function as a tumor suppressor.

## SUPPLEMENTARY DATA

Supplementary Data are available at NAR Online.

SUPPLEMENTARY DATA

## References

[1] Tanaka K. (2010). Multiple functions of the S-phase checkpoint mediator. Biosci. Biotechnol. Biochem..

[2] Liu S., Opiyo S.O., Manthey K., Glanzer J.G., Ashley A.K., Amerin C., Troksa K., Shrivastav M., Nickoloff J.A., Oakley G.G. (2012). Distinct roles for DNA-PK, ATM and ATR in RPA phosphorylation and checkpoint activation in response to replication stress. Nucleic Acids Res..

[3] Feijoo C. (2001). Activation of mammalian Chk1 during DNA replication arrest: a role for Chk1 in the intra-S phase checkpoint monitoring replication origin firing. J. Cell Biol..

[4] Zuazua-Villar P., Rodriguez R., Gagou M.E., Eyers P.A., Meuth M. (2014). DNA replication stress in CHK1-depleted tumour cells triggers premature (S-phase) mitosis through inappropriate activation of Aurora kinase B. Cell Death Dis..

[5] Ciccia A., Elledge S.J. (2010). The DNA damage response: making it safe to play with knives. Mol. Cell.

[6] Myriam Cuadrado B.M.-P., Murga M., Toledo L.I., Gutierrez-Martinez P., Lopez E., Fernandez-Capetillo O. (2006). ATM regulates ATR chromatin loading in response to DNA double-strand breaks. JME.

[7] Bartek J., Bartkova J., Lukas J. (2007). DNA damage signalling guards against activated oncogenes and tumour progression. Oncogene.

[8] Smith J., Tho L.M., Xu N.H., Gillespie D.A. (2010). The ATM-Chk2 and ATR-Chk1 pathways in DNA damage signaling and cancer. Adv. Cancer Res..

[9] Yamane A., Robbiani D.F., Resch W., Bothmer A., Nakahashi H., Oliveira T., Rommel P.C., Brown E.J., Nussenzweig A., Nussenzweig M.C. (2013). RPA accumulation during class switch recombination represents 5′-3′ DNA-end resection during the S-G2/M phase of the cell cycle. Cell Reports.

[10] Marechal A., Li J.M., Ji X.Y., Wu C.S., Yazinski S.A., Nguyen H.D., Liu S., Jimenez A.E., Jin J., Zou L. (2014). PRP19 transforms into a sensor of RPA-ssDNA after DNA damage and drives ATR activation via a ubiquitin-mediated circuitry. Mol. Cell.

[11] Zou L. (2003). Sensing DNA damage through ATRIP recognition of RPA-ssDNA complexes. Science.

[12] Kumagai A., Dunphy W.G. (2000). Claspin, a novel protein required for the activation of Chk1 during a DNA replication checkpoint response in xenopus egg extracts. Mol. Cell.

[13] Melo J., Toczyski D. (2002). A unified view of the DNA-damage checkpoint. Curr. Opin. Cell Biol..

[14] Liu S., Song N., Zou L. (2012). The conserved C terminus of Claspin interacts with Rad9 and promotes rapid activation of Chk1. Cell Cycle.

[15] Mailand N., Bekker-Jensen S., Bartek J., Lukas J. (2006). Destruction of Claspin by SCFbetaTrCP restrains Chk1 activation and facilitates recovery from genotoxic stress. Mol. Cell.

[16] Zhang D., Zaugg K., Mak T.W., Elledge S.J. (2006). A role for the deubiquitinating enzyme USP28 in control of the DNA-damage response. Cell.

[17] Faustrup H., Bekker-Jensen S., Bartek J., Lukas J., Mailand N. (2009). USP7 counteracts SCFbetaTrCP- but not APCCdh1-mediated proteolysis of Claspin. J. Cell Biol..

[18] Lin S.Y., Li K., Stewart G.S., Elledge S.J. (2004). Human Claspin works with BRCA1 to both positively and negatively regulate cell proliferation. Proc. Natl. Acad. Sci. U.S.A..

[19] Sato K., Sundaramoorthy E., Rajendra E., Hattori H., Jeyasekharan A.D., Ayoub N., Schiess R., Aebersold R., Nishikawa H., Sedukhina A.S. (2012). A DNA-damage selective role for BRCA1 E3 ligase in claspin ubiquitylation, CHK1 activation, and DNA repair. Curr. Biol..

[20] Bekker-Jensen S., Rendtlew Danielsen J., Fugger K., Gromova I., Nerstedt A., Lukas C., Bartek J., Lukas J., Mailand N. (2010). HERC2 coordinates ubiquitin-dependent assembly of DNA repair factors on damaged chromosomes. Nat. Cell Biol..

[21] Danielsen J.R., Povlsen L.K., Villumsen B.H., Streicher W., Nilsson J., Wikstrom M., Bekker-Jensen S., Mailand N. (2012). DNA damage-inducible SUMOylation of HERC2 promotes RNF8 binding via a novel SUMO-binding Zinc finger. J. Cell Biol..

[22] Izawa N., Wu W., Sato K., Nishikawa H., Kato A., Boku N., Itoh F., Ohta T. (2011). HERC2 Interacts with Claspin and regulates DNA origin firing and replication fork progression. Cancer Res..

[23] Berthouze M., Venkataramanan V., Li Y., Shenoy S.K. (2009). The deubiquitinases USP33 and USP20 coordinate beta2 adrenergic receptor recycling and resensitization. EMBO J..

[24] Han S.O., Xiao K., Kim J., Wu J.H., Wisler J.W., Nakamura N., Freedman N.J., Shenoy S.K. (2012). MARCH2 promotes endocytosis and lysosomal sorting of carvedilol-bound beta(2)-adrenergic receptors. J. Cell Biol..

[25] Li Z., Wang D., Messing E.M., Wu G. (2005). VHL protein-interacting deubiquitinating enzyme 2 deubiquitinates and stabilizes HIF-1alpha. EMBO Reports.

[26] Yee Koh M., Spivak-Kroizman T.R., Powis G. (2008). HIF-1 regulation: not so easy come, easy go. Trends Biochem. Sci..

[27] Xu X., Stern D.F. (2003). NFBD1/KIAA0170 is a chromatin-associated protein involved in DNA damage signaling pathways. J. Biol. Chem..

[28] Yuan J., Luo K., Zhang L., Cheville J.C., Lou Z. (2010). USP10 regulates p53 localization andstability by deubiquitinating p53. Cell.

[29] Attwooll C.L., Akpinar M., Petrini J.H. (2009). The mre11 complex and the response to dysfunctional telomeres. Mol. Cell. Biol..

[30] Lindsey-Boltz L.A., Sercin O., Choi J.H., Sancar A. (2009). Reconstitution of human claspin-mediated phosphorylation of Chk1 by the ATR (ataxia telangiectasia-mutated and rad3-related) checkpoint kinase. J. Biol. Chem..

[31] Shanmugam I., Abbas M., Ayoub F., Mirabal S., Bsaili M., Caulder E.K., Weinstock D.M., Tomkinson A.E., Hromas R., Shaheen M. (2014). Ubiquitin specific peptidase 20 regulates Rad17 stability, checkpoint kinase 1 phosphorylation and DNA repair by homologous recombination. J. Biol. Chem.

[32] Wu W., Sato K., Koike A., Nishikawa H., Koizumi H., Venkitaraman A.R., Ohta T. (2010). HERC2 is an E3 ligase that targets BRCA1 for degradation. Cancer Res..

[33] Martín Y., Cabrera E., Amoedo H., Hernández-Pérez S., Domínguez-Kelly R., Freire R. (2014). USP29 controls the stability of checkpoint adaptor Claspin by deubiquitination. Oncogene.

[34] Liu C., Wu J., Paudyal S.C., You Z., Yu X. (2013). CHFR is important for the first wave of ubiquitination at DNA damage sites. Nucleic Acids Res..

[35] Seshagiri S., Stawiski E.W., Durinck S., Modrusan Z., Storm E.E., Conboy C.B., Chaudhuri S., Guan Y., Janakiraman V., Jaiswal B.S. (2012). Recurrent R-spondin fusions in colon cancer. Nature.

[36] Cancer Genome Atlas Research N. (2013). Comprehensive molecular characterization of clear cell renal cell carcinoma. Nature.

[37] Weinstein J.N., Akbani R., Broom B.M., Wang W., Verhaak R.G., McConkey D., Lerner S., Morgan M., Creighton C.J., Smith C. (2014). Comprehensive molecular characterization of urothelial bladder carcinoma. Nature.

