# Supplementary material for: HERC2/USP20 coordinates CHK1 activation by modulating CLASPIN stability
Source: Nucleic Acids Res. 2014 Oct 17;42(21):13074–81. doi: 10.1093/nar/gku978 (PMC4245974; doi:10.1093/nar/gku978)
Supplement: SUPPLEMENTARY DATA [file supp_42_21_13074__index.html]

HERC2/USP20 coordinates CHK1 activation by modulating CLASPIN stability — HERC2/USP20 coordinates CHK1 activation by modulating CLASPIN stability — SUPPLEMENTARY DATA 

# HERC2/USP20 coordinates CHK1 activation by modulating CLASPIN stability

## SUPPLEMENTARY DATA

**Files in this Data Supplement:**

- SUPPLEMENTARY DATA
