# Supplementary material for: HERC2/USP20 coordinates CHK1 activation by modulating CLASPIN stability
Source: Nucleic Acids Res. 2014 Oct 17;42(21):13074–81. doi: 10.1093/nar/gku978 (PMC4245974; doi:10.1093/nar/gku978)
Supplement: SUPPLEMENTARY DATA [file supp_gku978_nar-01817-x-2014-File007.pdf]

Zhu et al., Supplemental Figures

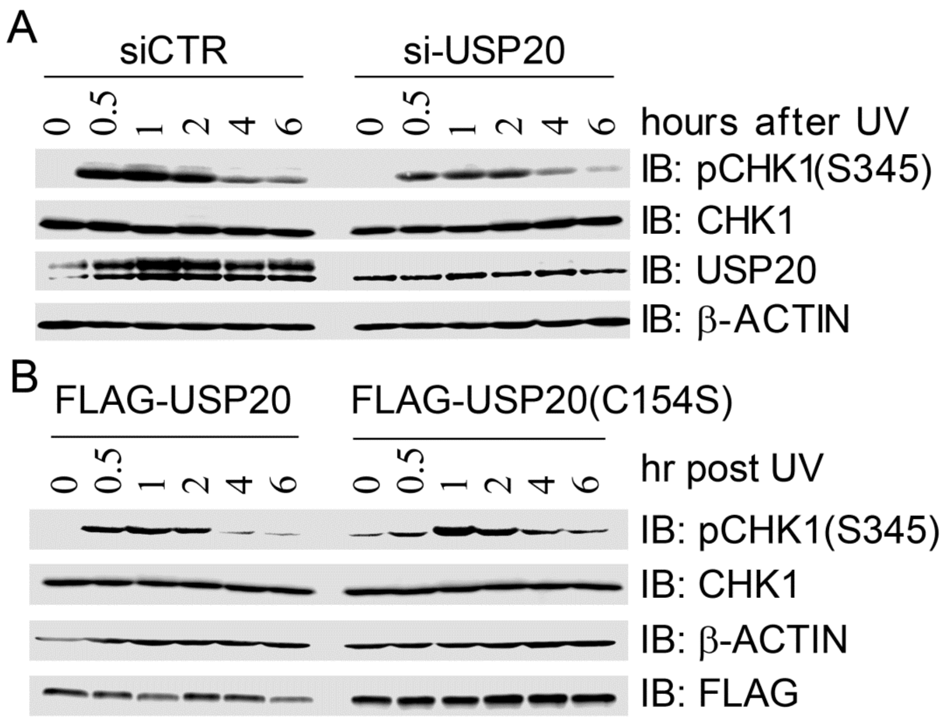

Figure S1. USP20 promotes CHK1 activation in response to UV radiation. (A) Depletion of USP20 delayed UV-induced CHK1 activation. 293T cells were transfected with a control siRNA (siCTR) or a USP20-specific siRNA (si1-USP20), cells were treated with UV (20 J/m^2) two days after transfection. Total cell lysates were harvested at different time points after UV irradiation and used for immuno-blotting with antibodies as indicated. The efficiency of USP20 knockdown was determined by immuno-blotting with antibodies against USP20 and β-actin. (B) Over-expression of the catalytic inactive mutant USP20(C154S) attenuated UV-induced CHK1 activation. 293T cells were transfected with FLAG-USP20 or FLAG-USP20(C154S). Cells were treated two days later with UV (20 J/m^2). Total cell lysates were harvested at different time points after UV irradiation and used for immuno-blotting with antibodies as indicated.

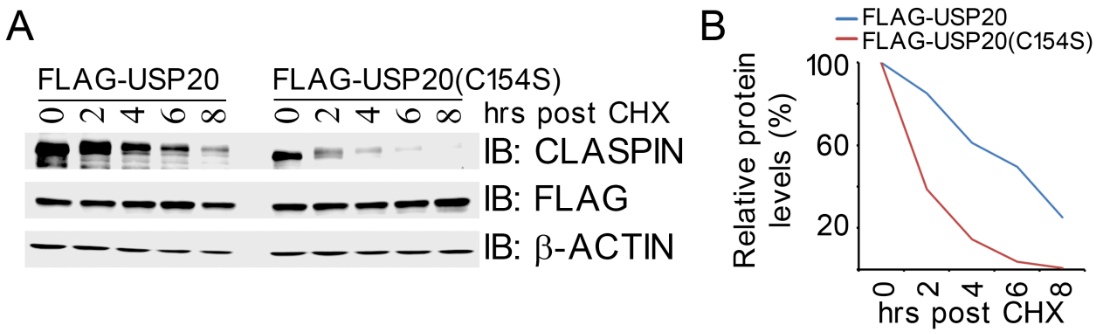

Figure S2. Overexpression of USP20(C154S) compromised CLASPIN stability. 293T cells were transfected with FLAG-USP20 or catalytic inactive mutant FLAG-USP20(C154S). Cells were treated two days later with cycloheximide (CHX), total cell lysates were harvested at different time points after CHX treatment and used for immunoblotting with antibodies as indicated (A). Quantification of the CLAPSIN/β-ACTIN ratio was plotted in (B).

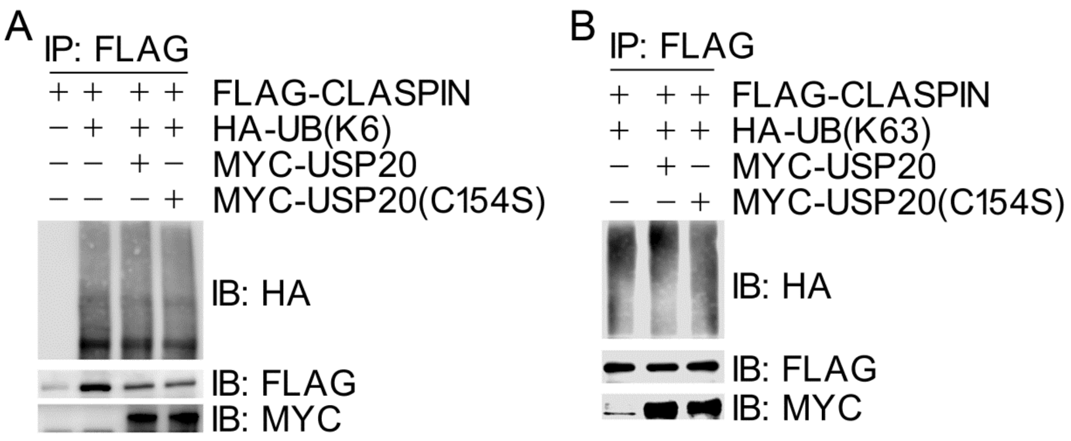

Figure S3. USP20 failed to deubiquitinate K6- or K63-linked polyubiquitination of CLASPIN. 293T cells were transfected with FLAG-CLASPIN, HA-UB(K6) (All the lysine residues except K6 were mutated to R) or HA-UB(K63) (All the lysine residues except K63 were mutated to R), and MYC-USP20 or MYC-USP20(C154S). Total cell lysates were harvested two days later and used for immunoprecipitation and immunoblotting with antibodies as indicated.

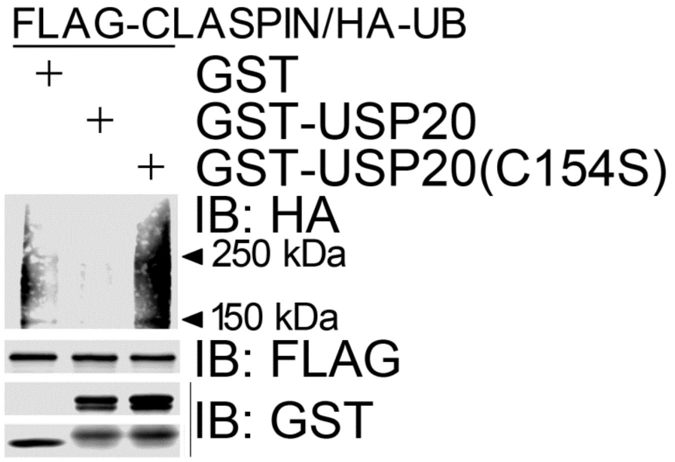

Figure S4. In vitro deubiquitination of CLASPIN by USP20. 293T cells were co-transfected with FLAG-CLASPIN and HA-UB. Total cell lysates were harvested and subjected to immunoprecipitation with an anti-FLAG antibody. The FLAG-USP20 immunocomplex was incubated with bacterially produced GST, GST-USP20, or GST-USP20(C154S) in the in vitro deubiquitination assay.

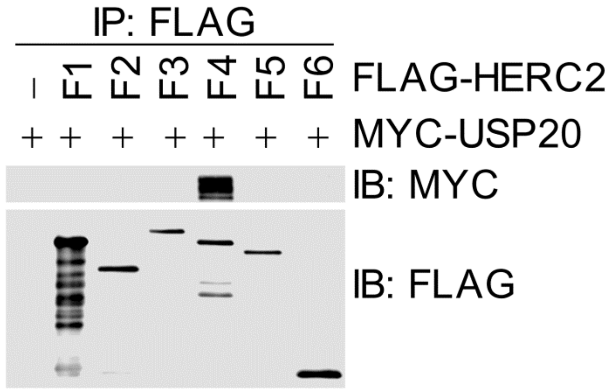

Figure S5. The F4 fragment of HERC2 (2600-3600 AAs), which contains ZZ domain, mediated the interaction with USP20. Total cell lysates extracted from 293T cells co-transfected with MYC-USP20 and FLAG-HERC2 or its fragments were subjected to immunoprecipitation with an anti-FLAG antibody followed by immunoblotting with an anti-MYC antibody.

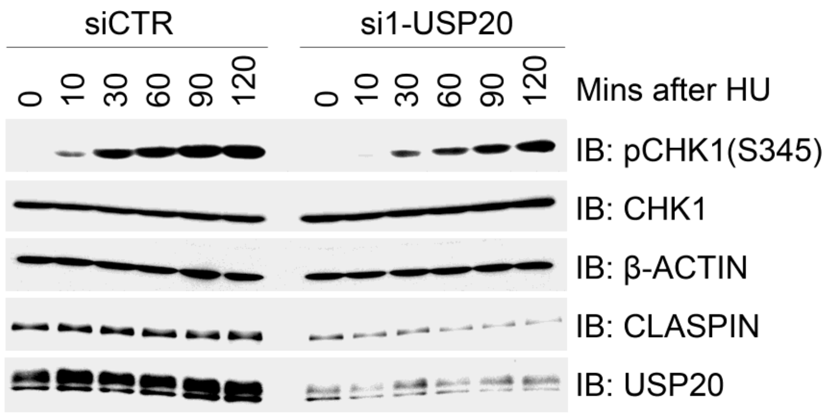

Figure S6. USP20 regulates CLASPIN stability after HU treatment. 293T cells were transfected with a control siRNA (siCTR) or a USP20-specific siRNA (si1-USP20), cells were treated with HU two days after transfection. Total cell lysates were harvested at different time points after HU treatment and used for immuno-blotting with antibodies as indicated.

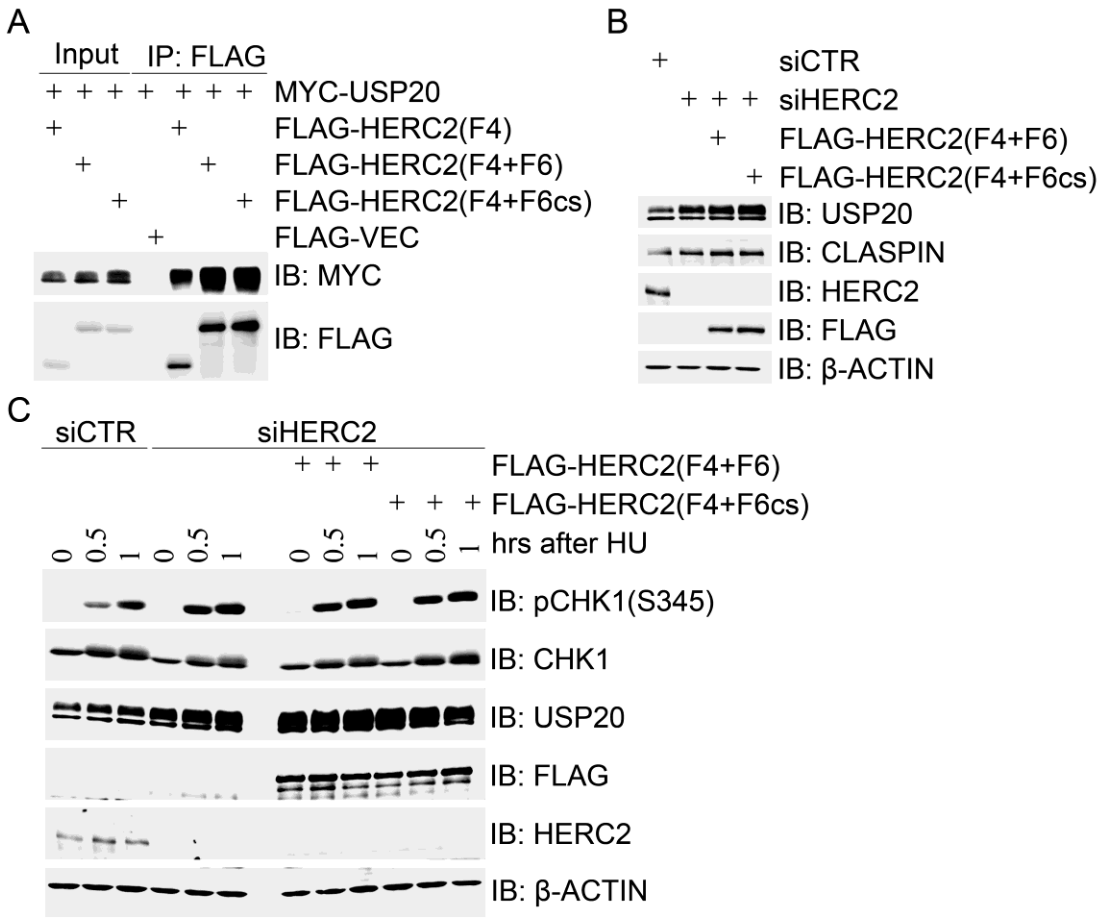

Figure S7. Expression of the fusion protein HERC2(F4+F6), which contains the HERC2/USP20 interaction domain and HERC2 catalytic domain, failed to rescue HERC2 depletion-induced USP20 stability and CHK1 activation after HU treatment. (A) HERC2(F4), HERC2(F4+F6), and HERC2(F4+F6CS) physically interacted with USP20. MYC-USP20 co-transfected with FLAG-HERC2(F4), FLAG-HERC2(F4+F6), or FLAG-HERC2(F4+F6CS). Total cell lysates were extracted 48 hours after transfection and subjected to immunoprecipitation and immunoblotting with antibodies as indicated. (B) Expression of HERC2(F4+F6) failed to rescue HERC2 depletion-induced USP20 stability. 293T cells were transfected with siCTR or si1-HERC2. Two days later, HERC2-depleted 293T cells were transfected with FLAG-HERC2(F4+F6) or FLAG-HERC2(F4+F6CS). Total cell lysates were harvested two days after the second transfection and subjected to immunoblotting with antibodies as indicated. (C) Expression of HERC2(F4+F6) failed to rescue HERC2 depletion-induced CHK1 activation after HU treatment. HERC2-depleted and HERC2(F4+F6)/HERC2(F4+F6cs)-expressing 293T cells were treated with HU at different time points as indicated. Total cell lysates were harvested and subjected to immunoblotting with antibodies as indicated.

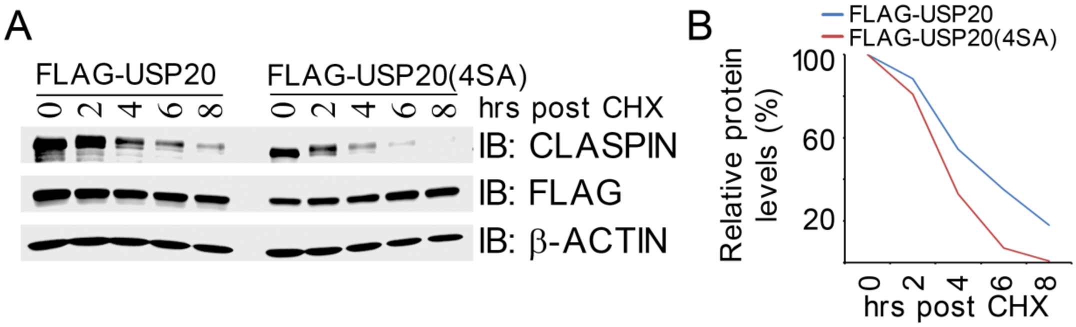

Figure S8. Overexpression of phosphorylation-deficient mutant USP20(4SA) compromised CLASPIN stability. 293T cells were transfected with FLAG-USP20

or phosphorylation-deficient mutant FLAG-USP20(4SA). Cells were treated two days later with CHX, total cell lysates were harvested at different time points after CHX treatment and used for immunoblotting with antibodies as indicated (A). Quantification of the CLAPSIN/β-ACTIN ratio was plotted in (B).

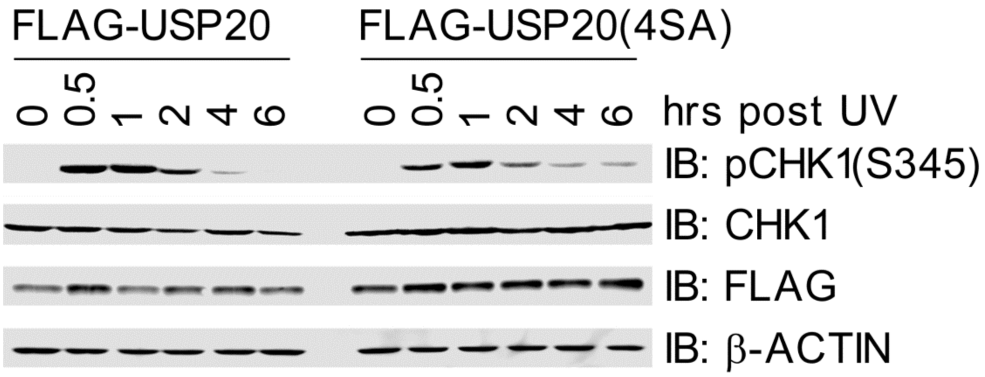

Figure S9. Phosphorylation-deficient mutant USP20(4SA) delayed UV-induced CHK1 activation. 293T cells expressing FLAG-USP20 or FLAG-USP20(4SA) were treated with UV (20 J/m^2) two days after transfection. Total cell lysates were harvested at different time points after UV irradiation and used for immuno-blotting with antibodies as indicated.

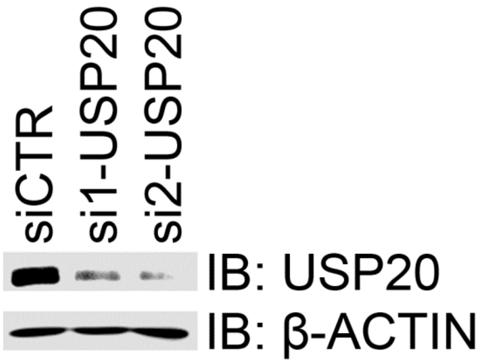

Figure S10. USP20 knockdown efficiency in A549 cells described in Figure 5A-5C.
